# Moderate Physical Training Ameliorates Cardiovascular Dysfunction Induced by High Fat Diet After Cessation of Training in Adult Rats

**DOI:** 10.3389/fphys.2019.00170

**Published:** 2019-03-12

**Authors:** Laize Peron Tófolo, Wilson Rinaldi, Adriane Barreto Gôngora, Camila Cristina Ianoni Matiusso, Audrei Pavanello, Ananda Malta, Douglas Lopes de Almeida, Tatiane Aparecida Ribeiro, Anna Rebeka Oliveira, Maria Natalia Chimirri Peres, James Andrew Armitage, Paulo Cezar de Freitas Mathias, Kesia Palma-Rigo

**Affiliations:** ^1^Laboratory of Secretion Cell Biology, Department of Biotechnology, Genetics and Cell Biology, State University of Maringá, Maringá, Brazil; ^2^Department of Physical Education, Faculty of Biomedical Sciences of Cacoal, Cacoal, Brazil; ^3^Department of Physical Education, State University of Maringá, Maringá, Brazil; ^4^Faculdade Adventista Paranaense, Ivatuba, Brazil; ^5^School of Medicine, Deakin University, Waurn Ponds, VIC, Australia

**Keywords:** high fat diet, moderate exercise, cardiovascular risk, cessation of training, detraining, cardiovascular variability, blood pressure, lipid profile

## Abstract

We aimed to test whether moderate physical training can induce long-lasting protection against cardiovascular risk factors induced by high fat diet (HFD) intake, even after cessation of training. 90-days-old Wistar rats were submitted to a sedentary lifestyle or moderate physical training, three times a week, for 30 days. Following this, at 120 days-of age, sedentary and trained rats received a hypercaloric diet (HFD) or a commercial diet normal fat diet (NFD) for 30 days. Body weight (BW) and food intake were evaluated weekly. At 150 days-of age, hemodynamic measures (systolic, diastolic, mean blood pressure, pulse pressure, pulse interval and heart rate) were made via an indwelling femoral artery catheter. Beat-to-beat data were analyzed to calculate power spectra of systolic blood pressure (SBP) and pulse interval. After euthanasia, mesenteric fat pads were removed and weighted and total blood was stored for later analysis of lipid profile. Consumption of a HFD increased blood pressure (BP), pulse pressure, low frequency BP variability, BW gain, fat pad stores and induced dyslipidemia. Interestingly, prior physical training was able to partially protect against this rise in BP and body fat stores. Prior physical training did not totally protect against the effects of HFD consumption but previously trained animals did demonstrate resistance to the development of cardiometabolic alterations, which illustrate that the benefits of physical training may be partially maintained even after 30 days of detraining period.

## Introduction

According to the World Health Organization (2018) non-communicable diseases such as cardiovascular disease, cancer, diabetes and chronic lung disease cause nearly two thirds of all deaths across the globe (Donaldson, 2017). In addition, cardiovascular disease is responsible for three quarters of the deaths in low and middle income countries, placing a burden on public health systems and emerging economies (World Health Organization, 2015). The etiology of cardiovascular disease is dependent on genetic and environmental factors, including sedentary lifestyle, and is exacerbated by metabolic disease such as hyperglycaemia, hyperlipidaemia, overweight, and obesity. Adding to the disease burden is the rapid change in dietary patterns across the globe, with an increase in palatable food intake, rich in high concentration of fat and sugar. Physical inactivity also contributes to a pattern of impaired health. Studies have shown that HFD consumption has a high correlation with metabolic and cardiovascular diseases, even when consumed for a short-term period (Barnes et al., 2003; Buettner et al., 2007; Gomes et al., 2013). Cardiovascular dysfunction in response to a HFD is thought to be dependent on sympathetic nervous system arousal that results in increased plasma norepinephrine turnover in the heart, increases renal sympathetic nerve activity, and increased BP variability (in the LF domain) contributing to the deregulation of BP (Prior et al., 2010; Armitage et al., 2012).

Wen and Wu (2012) suggest that a sedentary lifestyle (failure to perform even 15 to 30 min/days of brisk walking) may increase the risk of developing heart disease by 20–30% (Wen and Wu, 2012). In this context, the increase of physical activity and concomitant energy expenditure is an efficient strategy to prevent or treat cardiometabolic diseases, such as hypertension (Batacan et al., 2016). It is known that physical exercise promotes a reduction of obesity and amelioration of cardiometabolic dysfunctions by activation of lipid metabolism and modulation of inflammation (Ross et al., 2000; Lazzer et al., 2011). When physical exercise is tailored to maximize aerobic metabolism, it promotes fat mobilization, reduction of BP and other benefits on cardiovascular system function. In addition, exercise may promote activation or inhibition of brain areas that regulate ANS function (Chari et al., 2010), thereby decreasing some of the negative effects of HFD intake.

The effect of detraining on cardiovascular and metabolic systems is controversial. Some studies suggest that cardiometabolic adaptations induced by exercise persist for between 1 and 4 weeks after the detraining period (Marini et al., 2008; Lehnen et al., 2010; Agarwal et al., 2012; Tofolo et al., 2014). Other studies indicate that cardiometabolic adaptations induced by exercise are abolished after 2, 4, 5, or 10 weeks of detraining (Kemi et al., 2004; Bocalini et al., 2010; Carneiro-Junior et al., 2013; Kilic-Erkek et al., 2014). Therefore, although physical exercise benefits are often thought to be related with its continuity (Kilic-Erkek et al., 2016), exercise cessation or detraining may be associated with a partial or complete loss of the physical benefits obtained in the period of physical exercise (Mujika and Padilla, 2000). This discrepancy in literature can be explained by, and depends on, variability of the types of physical exercise (intensity and frequency of exercise), duration of exercise, detraining and, importantly the period of life when the exercise was performed (Waring et al., 2015).

Considering the evident deleterious effects of HFD and the controversial benefit of detraining in the cardiovascular system, the effect of HFD intake after cessation of moderate intensity-LF exercise in adult life is poorly understood. We hypothesized that moderate physical training performed three times per week for 30 days on adult life can have extended protective cardiometabolic alterations even after cessation of training in HFD exposed rats.

## Materials and Methods

### Experimental Model

One hundred and twenty male Wistar rats aged 85 days were housed (five animals per cage) and were provided with water and food *ad libitum* in a room maintained at 22 ± 2°C with a 12/12 h light/dark cycle. After 5 days of environmental adaptation, a group of 90-day-old rats underwent treadmill exercise training for 30 days (EXE group). The control group remained sedentary (SED group). During this period (90–120 days of life), all animals were fed a (NFD; AIN 93 M, Nuvital-Curitiba, PR 3.801 kcal/g). After this period (120–150 days of life), animals were fed with a NFD or high-fat-diet (HFD; hypercaloric home-made diet containing 35% lard; 5.817 kcal/g) and all remained sedentary. The composition of the NFD and HFD has been previously described (Barella et al., 2012). Thus, four groups were produced: sedentary (SED) and exercised (EXE) rats subjected exclusively to a NFD (SED-NFD and EXE-NFD, respectively) and SED and EXE rats subjected after training to a HFD (SED-HFD and EXE-HFD, respectively). During the training period, all animals were fed with the NFD. This study was carried out in accordance with the recommendations of National Council of Animal Experiments Control (CONCEA) and the Brazilian Society of Science in Laboratory Animals (SBCAL). The protocol was approved by the Ethics Committee of the State University of Maringá (protocol number 9213241014).

### Treadmill Physical Training Protocol

Animals were trained in a rodent treadmill (Panlab, Harvard Apparatus^®^, Cornellà- Barcelona – Spain) with electronic velocity control. Physical training was performed in the afternoon (around 14:00 p.m.), three times a week for 30 days (12 sessions from 90 to 120 days of life). The programming of physical training was modified from a moderate physical training protocol for rats previously described (Tofolo et al., 2014) and proposed by Negrão et al. (1992). The training intensity has been previously been confirmed using a maximal effort test, as per previous studies (Tofolo et al., 2014). The training protocol became more rigorous over time, as trained animals became more efficient at running, starting with sessions of 16 cm/sec for 10 min and finishing with sessions of 26 cm/sec for 60 min (Table 1).

**Table 1:** Moderate intensity physical training protocol.

Sessions	Speed (cm/sec)	Time (min)	Intensity corresponding to VO2max
1°	16	10	50%
2°	16	20	50%
3°	16	20	50%
4°	16	30	50%
5°	19	40	66%
6°	19	40	66%
7°	19	40	66%
8°	22	50	66%
9°	22	50	68%
10°	22	55	68%
11°	26	60	68%
12°	26	60	68%

### Biometric Parameters and Lipid Profile

A separate group of animals, from the four experimental groups, (not subject to surgical protocols) was also generated. Average food intake was measured 3 times a week from the first to eighth weeks of treatment. Food intake was calculated as the difference between the amount of food remaining and the total provided, which was divided by the number of days and the number of rats in the cage. As energetic values between the diets were different, food consumption in grams was converted into caloric intake. BW was measured once a week during the experimental period. At 150 days-of age rats were euthanized by decapitation and the mesenteric store removed and weighed, to measure body fat accumulation. A separate group of 28 animals, was used to evaluate visceral fat at 120 days of life. Visceral fat was presented relative to 100 g of BW. Blood samples were collected and plasma was used for measurement of total cholesterol, HDL cholesterol and triglycerides by the enzymatic method using a colorimetric commercial kit (Gold Analisa^®^, Belo Horizonte, Minas Gerais, Brazil). LDL and VLDL cholesterols were calculated according to the Friedewald equation: LDL = Total cholesterol – (HDL + VLDL) and VLDL = Triglycerides/5 (Berlanga et al., 2005).

### Surgery for Arterial Catheter Implantation

At 146-days of age, a subset of the animals, from all experimental groups, were anesthetized (intramuscular ketamine-xylazine; 3 and 0.6 mg/100 g of BW, respectively) and a P10 catheter (P10 cannula-Micro-Renathane) connected to a P50 cannula (ClearTygon) filled with heparinized saline (250 units/mL) was implanted into the femoral artery and advanced (4 cm) into the abdominal aorta. Doxycycline (2 mg/Kg of BW, intra-arterial) was administered during the surgery as prophylaxis against infection and Metamizole provided analgesia (12.5 mg/day in drinking water) for two days following surgery. During the post-surgery recovery period, animals were examined daily and those who showed signs of pain or stress were excluded from the study. Heparinized saline (250 units/mL) was injected through the cannula in the second and fourth day to maintain cannula patency (Martin et al., 1996). Post-surgery, animals were housed individually and cardiovascular recordings were performed 4 days after the surgery (Poppendieck et al., 2013).

### Blood Pressure Recordings

The experiments were performed after 1 h adaptation to the experimental room, always during the mid-afternoon period (14:00 p.m.). The full BP trace was recorded enabling systolic, diastolic, mean blood pressure, pulse pressure and heart rate to be derived. Baseline recordings were made in freely moving rats in their home cage over a 30 min period. The arterial cannula was connected to a fluid-filled BP transducer (MLT0699, AD Instruments, Dunedin, New Zealand), connected to a signal amplifier (Insight, Ribeirão Preto/SP Brazil). Recordings were sampled at 1000 Hz using an analog-to-digital converter board (CODAS, 1-kHz sampling frequency, Dataq Instruments, Inc., Akron, OH, United States). Analyses were performed on a beat-to-beat basis to quantify the changes in the BP and PI (Palma-Rigo et al., 2010).

### Cardiovascular Variability and Cardiac Baroreflex Sensitivity

Beat-to-beat data were analyzed to calculate power spectra of SBP and PI (a surrogate for the heart rate) using the CardioSeries v 2.4 software^1^. The power spectra was calculated for multiple overlapping (by 50%) segments of 512 data points (51.2 s) of 30 min stationary SBP and PI using a fast Fourier transform (Santos et al., 2012). The spectra were integrated in the (LF; 0.2 to 0.75 Hz) and (HF, 0.75 to 3.0 Hz) bands (Santos et al., 2012). The spontaneous cardiac baroreflex sensitivity was estimated by the sequence method, over 30 min, (Di Rienzo et al., 2010) in four consecutive beats in which increases in SBP were followed by PI lengthening (up sequence) and decreases in SBP was followed by PI shortening (down sequence), with a linear correlation higher than 0.85 (Silva et al., 2015).

### Statistical Analysis

Data are expressed as mean ± standard error of the mean (SEM). GraphPad Prism version 6.01 for Windows (GraphPadSoftware, La Jolla, CA, United States) was used for statistical analyses and plots. Statistical analyses were performed using two-way analysis of variance (ANOVA) followed by the Tukey multiple comparisons test. *P*-values < 0.05 were considered significant when analyzing the main effect of diet (*p_d_*), exercise (*p_e_*), their interaction (*p_i_*; diet vs. exercise) and the differences between groups.

## Results

### Food Intake and Body Weight Gain

**FIGURE 1:**
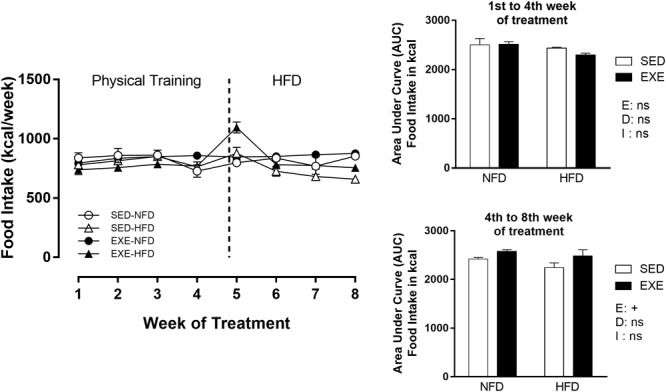
Food intake (Kcal/week) during 8 weeks of treatment. *n* = 15–20 per group. NFD, normal-fat diet; HFD, high-fat diet; SED, sedentary animals; EXE, exercised animals; I, interaction between exercise and diet factors; E, exercise factor; D, diet factor. ^+^*p* < 0.05 for probability based on a two-way analysis of variance.

During the physical training period, from 90 to 120 days of life, training did not affect food intake. At the cessation of the exercise period (120–150 days of life), the HFD and NFD EXE showed 8,6% increase (*p_e_* < 0.05) in food intake compared with HFD and NFD SED (Figure 1).

**FIGURE 2:**
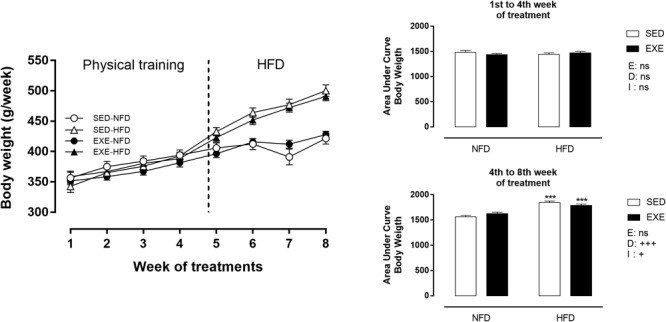
Body weight (BW) during 8 weeks of treatment. *n* = 17–20 per group. NFD, normal-fat diet; HFD, high-fat diet; SED, sedentary animals; EXE, exercised animals; I, interaction between exercise and diet factors; E, exercise factor; D, diet factor. ^+++^*p* < 0.001 and ^++^*p* < 0.01 for probability based on a two-way analysis of variance. ^∗∗∗^*p* < 0.001 statistical significance between NFD and HFD with 150-day-old, for the probability based on a Tukey multiple comparisons test.

Body weight did not change during the training period (Figure 2). After the training period (120–150 days of life), consumption of a HFD induced an increase in BW (sedentary: +18% and exercised: +10%; *p_d_* < 0.001), compared with NFD exposure (Figure 2). This increase was attenuated by 3.3% in EXE and resulted in a diet x exercise interaction (*p_i_* < 0.05). Sedentary and exercised NFD animals did not show any difference in BW over the experimental protocol.

### Fat Deposition and Soleus Muscle

**FIGURE 3:**
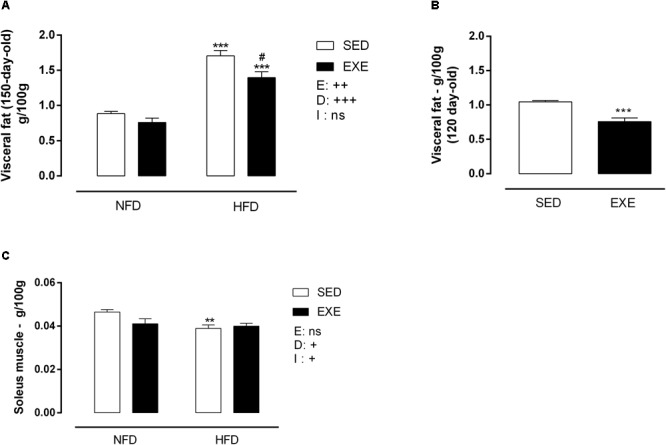
Body tissue weight of 120 and 150-day-old animals. **(A)** Visceral fat (g/100gBW) at 150-day-old, **(B)** visceral fat (g/100gBW) at 120 day-old animals, **(C)** soleus muscle (g/100gBW) at 150-day-old animals. *n* = 11–19 per group. NFD, normal-fat diet; HFD, high-fat diet; SED, sedentary animals; EXE, exercised animals; I, interaction between exercise and diet factors; E, exercise factor; D, diet factor. ^+++^*p* < 0.001, ^++^*p* < 0.01 and ^+^*p* < 0.05 for probability based on a two-way analysis of variance. ^∗∗∗^*p* < 0.001 and ^∗∗^*p* < 0.01 statistical significance between NFD and HFD with 150-day-old and ^#^*p* < 0.05 statistical significance between HFD groups, for the probability based on a Tukey multiple comparisons test.

At 150 days of life, HFD induced an increase in visceral fat pad deposition (sedentary: +93%, exercised: +83%; *p_d_* < 0.001) compared with NFD animals (Figure 3A). Interestingly, previous training induced a resistance to fat gain in HFD animals, resulting in an 18% decrease in fat pad mass (*p_e_* < 0.01). NFD fed sedentary and EXE did not show differences in fat deposition at 150-day-old. After the training period, at 120-days of life, EXE showed a 27% decrease in fat pad mass (*p* < 0.001) compared to 120-day-old SED (Figure 3B). After the cessation of the training, the reduction in fat deposition was maintained in 150-day-old EXE (*p* < 0.001) (Figure 3B).

Consumption of HFD induced a 16% decrease in soleus muscle mass in SED-HFD animals compared to SED-NFD animals *p* < 0.01 (Figure 3C). A diet x exercise interaction was observed, due to lower soleus muscle mass in exercised and HFD animals (*p_i_* < 0.05) (Figure 3C).

### Blood Pressure, Pulse Pressure and Heart Rate

**FIGURE 4:**
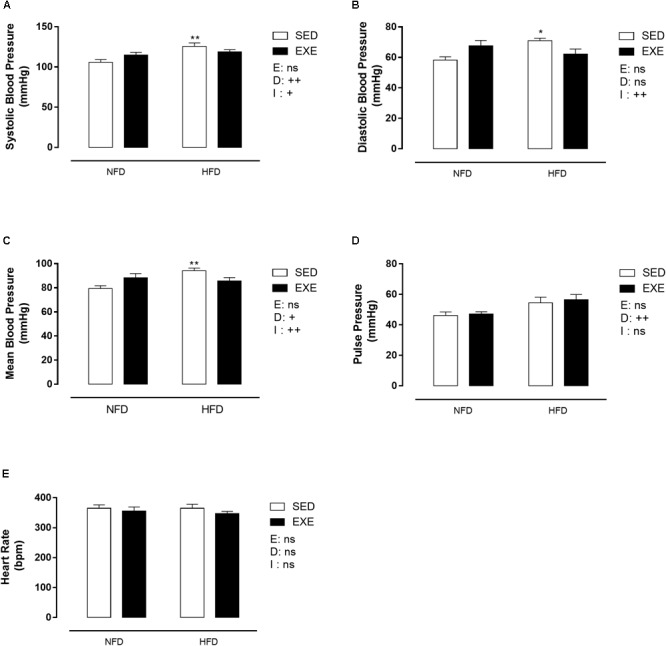
Blood pressure in 150-day-old animals. **(A)** Systolic blood pressure, **(B)** diastolic blood pressure, **(C)** mean blood pressure, **(D)** pulse pressure, **(E)** heart rate. *n* = 7–9 per group. NFD, normal-fat diet; HFD, high-fat diet; SED, sedentary animals; EXE, exercised animals; I, interaction between exercise and diet factors; E, exercise factor; D, diet factor. ^++^*p* < 0.01 and ^+^*p* < 0.05 for probability based on a two-way analysis of variance. ^∗∗^*p* < 0.01 and ^∗^*p* < 0.05 statistical significance of the differences between NFD and HFD for the probability based on a Tukey multiple comparisons test.

Figure 4A shows that SBP was elevated in SED-HFD compared with SED-NFD animals (19%; *p* < 0.01). However, no effect of the diet was observed in EXE-HFD animals, resulting in a significant effect of diet, (*p_d_* < 0.01) and a significant interaction between factors (*p_i_* < 0.05). A similar pattern was observed in mean BP (Figure 4C), with a 19% increase observed in SED-HFD animals compared with SED-NFD animals, (*p_d_* < 0.05) and an interaction (*p_i_* < 0.01). Diastolic blood pressure was increased in SED-HFD compared with SED-NFD animals (22%; *p* < 0.05), and a significant interaction between factors was noted (*p_i_* < 0.01, Figure 4B). Pulse pressure was 19% increased in HFD (*p_d_* < 0.01, Figure 4D) compared with NFD animals. There was no change in heart rate in any group (Figure 4E).

### Cardiovascular Variability and Cardiac Baroreflex Sensitivity

**FIGURE 5:**
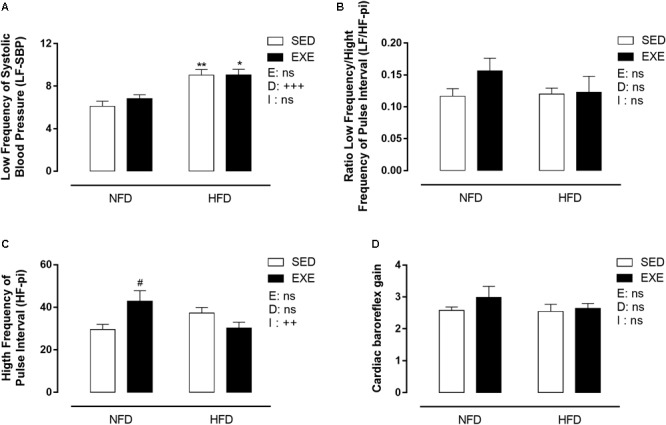
Spectral analyses on BP and pulse interval in 150-day-old animals. **(A)** Low frequency of systolic blood pressure, **(C)** high frequency of pulse interval, **(B)** ratio low frequency/ high frequency of pulse interval, **(D)** cardiac baroreflex gain. *n* = 5–9 per group. NFD, normal-fat diet; HFD, high-fat diet; SED, sedentary animals; EXE, exercised animals; I, interaction between exercise and diet factors; E, exercise factor; D, diet factor. ^+++^*p* < 0.001, ^++^*p* < 0.01 for probability based on a two-way analysis of variance. ^∗∗^*p* < 0.01 and ^∗^*p* < 0.05 statistical significance of the differences between NFD and HFD. ^#^*p* < 0.05 statistical significance of sedentary versus EXE for the probability based on a Tukey multiple comparisons test.

The LF power of SBP, an estimation of vascular sympathetic activity, was increased by 41% when consuming a HFD (*p_d_* < 0.001, Figure 5A). The LF/HF ratio of PI (LF/HF-PI), an estimation of cardiac sympathetic activity, was not affected by any factor (Figure 5B). The HF power of PI (HF-PI), an estimation of parasympathetic activity, was increased by 45% in EXE-NFD animals, compared with SED-NFD animals (*p* < 0.05, Figure 5C), with no effect in HFD animals, which lead to a significant interaction (*p_i_* < 0.01, Figure 5C). The cardiac baroreflex gain was not affected by any factor (Figure 5D).

### Lipid Profile

**FIGURE 6:**
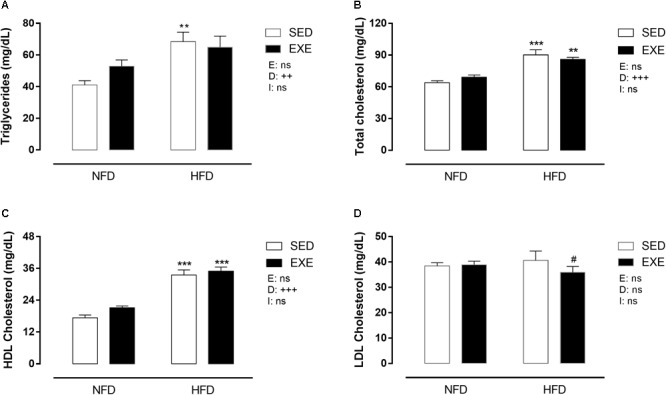
Lipid profile in 150-day-old animals. **(A)** Triglycerides, **(B)** total cholesterol, **(C)** HDL cholesterol, **(D)** LDL cholesterol. *n* = 8–10 per group. NFD, normal-fat diet; HFD, high-fat diet; SED, sedentary animals; EXE, exercised animals; I, interaction between exercise and diet factors; E, exercise factor; D, diet factor. ^+++^*p* < 0.001, ^++^*p* < 0.01 and ^+^*p* < 0.05 for probability based on a two-way analysis of variance. ^∗∗∗^*p* < 0.001 and ^∗∗^*p* < 0.01 statistical significance of the differences between NFD and HFD. ^#^*p* < 0.05 statistical significance of sedentary versus EXE for the probability based on a Tukey multiple comparisons test.

Figure 6 shows that HFD induced dyslipidemia in both, sedentary and trained groups. Triglyceride levels were increased in HFD fed animals (SED: +66% and EXE: +23%; *p_d_* < 0.001), compared with NFD feed animals (Figure 6A). HFD increased total cholesterol levels in sedentary and exercised rats (+41% and 24%, respectively), compared with NFD animals (*p_d_* < 0.001, Figure 6B). HDL cholesterol was increased by 79% in HFD fed animals (*p_d_* < 0.001, Figure 5C), compared with NFD feed animals. The LDL cholesterol was not affected by any factor (Figure 6D).

## Discussion

The current study shows, for the first time, that moderate physical training performed three times a week in adulthood protects against BP increase induced by HFD consumption even when the HFD occurs during a detraining period. It is well known that HFD consumption is strongly associated with increased cardiovascular risks, including hypertension, sympathetic over activity, tachycardia and dyslipidemia among other parameters (Barnes et al., 2003; Prior et al., 2010; Armitage et al., 2012; Tofolo et al., 2014; Xue et al., 2016). The current study used a physical training protocol that engaged aerobic improvement and ran for a sufficient time to stimulate positive cardiovascular alterations that persisted even after a period without physical training. Recently, it has been suggested that exercise at an intensity of between than 80–85% of VO_2_max may reduce the loss of the exercise effect even after a defined detraining period (Waring et al., 2015).

Cardiovascular homeostasis is maintained by different regulatory mechanisms, including the balance of sympathetic and parasympathetic activity to peripheral vasculature and the heart (Malliani et al., 1991). Other factors that may affect BP include the ingestion of hypercaloric foods or diets rich in fat, that contribute directly to an increase in adipose tissue accumulation and obesity development (Hall et al., 2000). In accordance with this, we observed that animals increased BW and fat pad stores when fed with a HFD. Moreover, sedentary HFD rats showed increased BP, which is well described in the literature (Barnes et al., 2003; Prior et al., 2010; Armitage et al., 2012). It has been suggested that HFD intake and obesity induce an imbalance in the ANS, with increase of sympathetic estimated by spectral analysis of LF of SBP (Van Vliet et al., 1995; Grassi et al., 2004; Prior et al., 2010; Armitage et al., 2012). Corroborating these findings, we showed that HFD intake for 30 days, induced elevated sympathetic vascular activity estimated by spectral analysis of LF-SBP. Furthermore, it has been shown that sympathetic activation to renal and skeletal muscle vasculature occurs in obese humans (Rumantir et al., 1999; Grassi et al., 2004).

The present benefit of previous exercise on BP of animals exposed to HFD appears to not depend on vascular sympathetic tone, as it is not reduced in EXE-HFD animals. This effect may depend on other factors, such as the inflammation driving increased BP. It has been observed in alternative models of obesity, induced by perinatal monosodium glutamate subcutaneous injection, that hypertension can be reduced by anti-inflammatory drugs, but via an alternative mechanism not dependent on increased cardiac sympathetic tone (da Cunha et al., 2011; Karlen-Amarante et al., 2012). The HFD promotes autonomic and inflammation-dependent hypertension, which is prevented by resistance training (Speretta et al., 2016). Interestingly, several mechanisms underlying the anti-inflammatory effects of exercise exist (Woods et al., 2006; Welty et al., 2016), and these are not dependent on autonomic modulation of immune system. Furthermore, it appears that muscle sympathetic vasoconstriction is balanced by metabolic vasodilatation promoted by exercise (Borresen and Lambert, 2008) and it is not well known how this balance changes during detraining.

The current study showed that a moderate physical training protocol performed three times a week in adult rats is effective in protect against the increase of BP induced by HFD intake, even after 30 days of detraining. Waring et al. (2015) showed that four weeks of vigorous physical exercise induced cardiac adaptations such as improved cardiac function, increased ventricular hypertrophy and increased maximal capacity of exercise, however, cessation of exercise quickly lead to regression of exercise induced benefices (Waring et al., 2015). The authors question if the adaptations gained from a lower intensity exercise training would be lost as rapidly due the lower impact of the stimulus. We show that our lower intensity exercise training protocol induced protection even 30 days after cessation of training; this pattern may depend on the low influence of the present physical exercise on the organism, which would be easier to deal facing the detraining.

In addition the current study shows an increase in parasympathetic cardiac activity in exercised NFD feed animals (estimated by spectral analysis in HF domain of the PI). Previous studies have reported an increase in parasympathetic activity induced by different types of exercise such as resistance training, high intensity interval training, aerobic training and swimming (Silveira et al., 2011; Barbosa Neto et al., 2013; Paolillo et al., 2014). It is possible that our exercised HFD animals demonstrate enhanced cardiovascular benefits induced by the training protocol. Corroborating with this research, Mostarda et al. (2009) showed that cardiac parasympathetic activity (estimated by HF spectral analysis of PI) was increased and maintained during a 3 week detraining period following moderate exercise, in diabetic rats (Mostarda et al., 2009). The imbalance of cardiac autonomic activity, characterized by an increase in sympathetic and decrease in parasympathetic activity, may initiate other cardiovascular complications, including hypertension, and pulse pressure increase. Moreover, when it is associated with BW and fat body increase and deregulation of lipid profile, it represents an important risk for cardiovascular disease and death (Tentolouris et al., 2006).

Pulse pressure, mathematically obtained by difference between systolic and diastolic blood pressure, is directly related to heart rate, ejection fraction, arterial stiffness and early dampening of the pulse wave. The present results show that HFD increased pulse pressure and exercise was unable to prevent this increase. Studies have considered that pulse pressure is a determinant of cardiovascular risk, mainly in elderly and obesity (Sousa et al., 2004; de la Sierra, 2006; Temelkova-Kurktschiev et al., 2009; Sikiru and Okoye, 2013), which may depend on the rigidity of large arteries, especially the aorta (Randall et al., 2005; De Pergola et al., 2012). Furthermore, it has been shown that both aging and high-fat diet increase aortic stiffness in B6D2F1 mice, which may depend on greater collagen, reduced elastin, and greater advanced glycation end products (Henson et al., 2014). Thus, the present increase in pulse pressure observed in HFD animals may depend on large artery rigidity induced by the diet.

HFD lead to dyslipidemia in both sedentary and exercised rats, with increased levels of total cholesterol and triglycerides, which are biomarkers of cardiovascular risk. This increase induced by HFD is well documented in the literature and appears to depend on disorders of lipid metabolism (Barella et al., 2015; Karupiah and Ismail, 2015; Sour et al., 2015; Kaprinay et al., 2016). LDL cholesterol, another biomarker for cardiovascular disease, but related with chronic sequelae such as atherosclerotic plaque development, is not affected by the 30 days of HFD consumption. Conversely, Chengyan Zhou et al. (2015) showed that 5, 11, and 18 weeks of HFD consumption induced an increase in LDL and decrease on HDL cholesterol (Zhou et al., 2015). We observed an increase in HDL cholesterol in HFD animals. It is known that rodents may demonstrate paradoxical imbalances in HDL and LDL cholesterol handling when fed HFD. This may be related to the increased total cholesterol in HFD animals and unchanged LDL cholesterol, suggesting that the excess of cholesterol is not mobilized by the tissues, leaving more cholesterol to be metabolized by the liver.

Interestingly, although cardiometabolic complications are strongly associated with HFD consumption it is important to emphasize that our animals showed a decrease in HFD intake, that may have been related to composition and palatability of the HFD which increased satiety (Corbit and Stellar, 1964). Consistent with our study, HFD rats had an increase of BW, even when their energetic intake as not increased. This pattern may be related to the diet composition, which is composed by a high amount of lipids and induces the onset of obesity (Sclafani, 2001; Borst and Conover, 2005). Furthermore, although the detraining is considered a sensitive period by some authors as a factor that stimulates the development of obesity (Shepard et al., 2001; Petibois et al., 2004) our animals did not show increase in BW or body fat mass.

In summary, HFD intake was responsible for inducing an increase in BP, in pulse pressure, in vascular sympathetic activity, in BW gain and fad pat stores, and dyslipidemia, which are all risk factors for cardiovascular diseases and death. Thirty days of moderate physical training performed three times a week promoted a protective effect on BP, LDL cholesterol and body fat against deregulation by HFD intake, even after 30 days of detraining. In conclusion, the moderate physical training, performed three times a week, for 30 days, promoted protection effect against cardiometabolic alterations induced by HFD, suggesting that physical training benefits on cardiovascular dysfunction in adult life may be maintained after a detraining period.

## Data Availability

The datasets generated for this study are available on request to the corresponding author.

## Author Contributions

LT contributed to the design of the study, acquisition, analysis and interpretation of data, and drafted the manuscript. WR, AG, CM, AP, AM, DdA, TR, AO, and MP contributed with the acquisition, revised critically the draft for important intellectual content. JA participated in the interpretation of the data for the work, revised critically the draft for important intellectual content. PM and KP-R conceived the study and participated in its design and coordination, revised critically the draft for important intellectual content. All authors gave final approval of the version to be published and agreed to be accountable for all aspects of the work.

## Conflict of Interest Statement

The authors declare that the research was conducted in the absence of any commercial or financial relationships that could be construed as a potential conflict of interest.

## Footnotes

1 http://www.danielpenteado.com/

## Abbreviations

ANS: Autonomic nervous system
BP: Blood pressure
BW: Body weight
EXE: exercised animals
HDL: High density lipoprotein
HF: High frequency
HFD: high fat diet
LDL: Low density lipoprotein
LF: Low frequency
NFD: Normal fat diet
PI: Pulse interval
SBP: Systolic blood pressure
SED: sedentary animals
VLDL: very low density lipoprotein
VO_2max_: maximal oxygen consumption during exercise
